# Mantle cell lymphoma patients in first relapse: we pretty much know what to do

**DOI:** 10.18632/oncotarget.27980

**Published:** 2021-08-17

**Authors:** Francesca Maria Quaglia, Carlo Visco

**Keywords:** mantle cell lymphoma, relapsed-refractory MCL, POD, ibrutinib, treatment algorithm

***Comment on:** Visco C, et al. Outcomes in first relapsed-refractory younger patients with mantle cell lymphoma: results from the MANTLE-FIRST study. Leukemia. 2021; 35:787–95. https://doi.org/10.1038/s41375-020-01013-3. [PubMed]*

Treatment of relapsed mantle cell lymphoma (MCL) patients is challenging. Disease behavior has typically been described to be largely heterogeneous [1]. The MANTLE-FIRST study, the first patient-level analysis of outcomes of relapsed-refractory (r/r) MCL after rituximab and cytarabine containing induction therapy, has clearly divided patients’ outcome depending on time to first relapse (early- *versus* late-POD) [2, 3]. While the latter experienced good expectations in terms of survival irrespective of the second line treatment, early-POD patients had significantly better outcome when treated with ibrutinib. This study, together with several independent observations [4, 5], has established ibrutinib as the new standard second line therapy in early-POD patients.

For late-POD patients the impact of treatment choice still needs to be clarified. In our study [2] bendamustine-based regimen (rituximab-bendamustine, R-B, and rituximab-bendamustine plus cytarabine, R-BAC) conferred similar survival rates to ibrutinib in this subgroup. We consider R-B, R-BAC or VR-CAP (bortezomib, rituximab, cyclophosphamide, doxorubicin, and prednisone) as reasonable time limited-treatment choice for patients with prior chemo-sensitive disease. However, given the expected response duration to BTKi in second line setting [5] and the generally favorable BTKi side effect profile relative to chemo-immunotherapy (CIT), unless major contraindications, we consider ibrutinib as standard second line therapy at any age.

The predictive significance of early-POD following less intensive frontline treatment, such as bendamustine-based induction regimen, is not well established. A recent work has shown shorter duration of first remission to be associated with inferior survival after both intensive and less intensive frontline therapy in a large retrospective cohort [6]. The BCL2-i venetoclax has been used *off-label* in this setting and could be a reasonable treatment option in the future.

Based on data showing that the efficacy of ibrutinib is greater when used early in treatment sequence [5], chemotherapy-sparing approaches such as BTKi plus anti-CD20 plus-minus venetoclax in treatment naïve MCL patients are under evaluation in clinical trials.

Early-POD younger patients (i.e., < 65) represent a high-risk population (10–20% of new diagnosed patients with MCL, overall) where allogeneic stem cell transplant (allo-SCT) appears as a valid option. Whenever achieving at least a good partial response or preferably a complete response (chemo-sensitive relapse), these patients have experienced good long-term outcome after allo-SCT [7]. Chimeric antigen receptor T-cells (CAR-T) may instead represent a unique opportunity for those patients that fail to achieve a substantial response after second line treatment [7, 8].

Unfortunately, despite the considerable clinical response to BTKi, resistance inevitably emerges [9]. When patients relapse on BTKi, disease is often aggressive and resistant to subsequent therapies. In this setting, CAR-T or clinical trials represent the mainstay for the future. A judicious evaluation of patients’ fitness and age is mandatory for subsequent treatment choice. CAR-T have held impressive short-term results in patients with MCL, and an update of the follow up is eagerly awaited [8].

In retrospective studies [9], R-BAC and venetoclax demonstrated a high response rate in the post-BTKi setting and were sometimes effective bridge to allo-SCT [10]. We have sufficient reasons (mutuated from chronic lymphocytic leukemia) to believe that patients that withhold BTKi due to intolerance (and not progression) might be a more favorable population. Among others, the non-covalent highly selective LOXO-305, which delivers consistently high target coverage regardless of BTK turnover rate, has been shown to have important efficacy in BTKi refractory patients, and in the presence of C481 acquired resistance mutation.

Overall, The MANTLE-FIRST study had the merit of comparing real-life second line most utilized approaches and supported time to POD as a robust independent prognostic marker in patients at first relapse [2]. The lack of biological characterization of included patients is one of the limitations of the study. Whether patients with early-POD or disease progression under BTKi are enriched with adverse biologic features (i.e., *TP53* mutations or deletions) require further clarification. To decipher the complex molecular network predicting refractoriness to CIT and BTKi - taking into account driver gene mutations, the B-cell receptor (BCR) pathway status of activation, and genetic and epigenetic determinants responsible for tumor cell evasion from BCR dependence - is the object of the ongoing FIL MANTLE-FIRST BIO study (https://clinicaltrials.gov/ number, *NCT04882475*).

Our proposal for a treatment algorithm with a perspective view on MCL management is depicted in Figure 1. We pretty much know what to do, but there is a clear need to identify biological subgroups associated with different clinical course. This is a critical next step to improve therapy allocation of patients who are r/r after cytarabine containing induction regimens and to guide the selection and optimal timing of new treatments with innovative approaches, including CAR-T, obtaining successful and durable responses.

**Figure 1:**
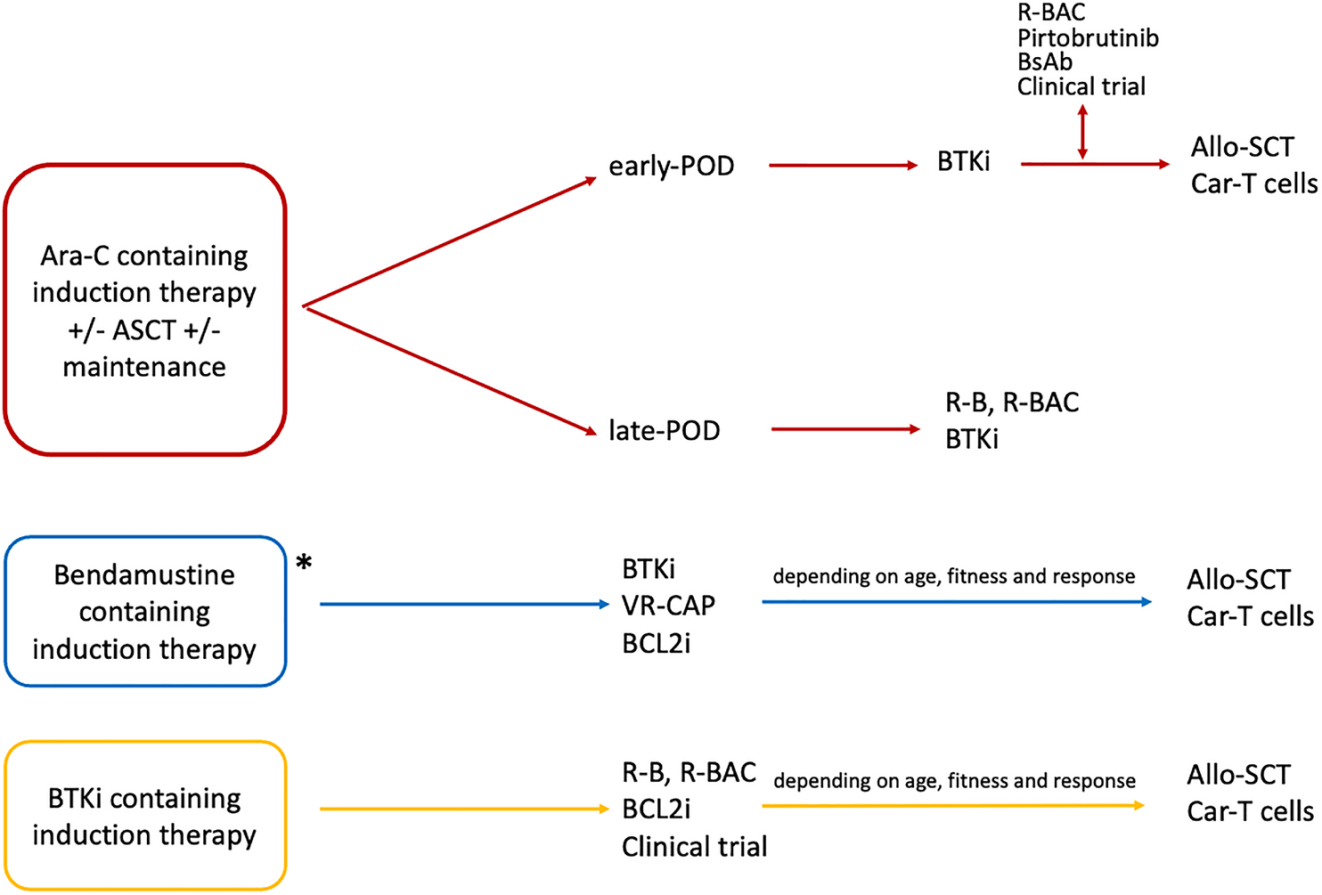
Proposal for approaching Mantle Cell Lymphoma (MCL) at first relapse. Allo-SCT: allogeneic stem cell transplant; Ara-C: cytarabine; ASCT: autologous stem cell transplant; BCL2i: BCL2-inhibitors (i.e., venetoclax); BsAb: monoclonal bispecific antibodies (i.e. mosunetuzumab, glofitamab, REGN1979); BTKi: Bruton tyrosine kinase inhibitors (i.e., ibrutinib, acalabrutinib, or zanubrutinib); CAR-T cells: chimeric antigen receptor T-cells; Pirtobrutinib: LOXO-305; POD: progression of disease; R-B: rituximab and bendamustine; R-BAC: rituximab and bendamustine plus cytarabine; VR-CAP: bortezomib plus rituximab, cyclophosphamide, doxorubicin, and prednisone. *Anthracycline containing induction (R-CHOP/VR-CAP) plus maintenance behave like late-POD younger patients.

## References

[1] Dreyling M, et al. Ann Oncol. 2017; 28:iv62–71. 10.1093/annonc/mdx223. 28881919

[2] Visco C, et al. Leukemia. 2021; 35:787–95. 10.1038/s41375-020-01013-3. 32782382

[3] Visco C, et al. Br J Haematol. 2019; 185:940–44. 10.1111/bjh.15643. 30407625

[4] Wang ML, et al. Blood. 2015; 126:739–45. 10.1182/blood-2015-03-635326. 26059948 PMC4528064

[5] Rule S, et al. Haematologica. 2018; 104:e211–14. 10.3324/haematol.2018.205229. 30442728 PMC6518912

[6] Bond DA, et al. Blood. 2019; 134:753. 10.1182/blood-2019-128415.

[7] Marangon M, et al. Cancers. 2021; 13:291. 10.3390/cancers13020291. 33466784 PMC7830938

[8] Wang M, et al. N Engl J Med. 2020; 382:1331–42. 10.1056/NEJMoa1914347. 32242358 PMC7731441

[9] McCulloch R, et al. Br J Haematol. 2020; 189:684–88. 10.1111/bjh.16416. 32011729

[10] Eyre TA, et al. Haematologica. 2019; 104:e68–71. 10.3324/haematol.2018.198812. 30190341 PMC6355471

